# Report of Henoch-Schönlein purpura associated with trastuzumab emtansine

**DOI:** 10.3332/ecancer.2024.1732

**Published:** 2024-07-30

**Authors:** Santiago Leandro Escobar-Dávila, Giovanna Patricia Rivas-Tafurt, Luis Álvaro Melo-Burbano, Luis Miguel Osorio-Toro, Edith Norela Benítez-Escobar, Duván Arley Galindes-Casanova, Jorge Hernán Izquierdo-Loaiza, Rodrigo Andrés Cárdenas-Perilla, Carlos Orozco-de la Hoz

**Affiliations:** 1 Facultad de Salud, Especialización en Medicina Interna, Universidad Santiago de Cali, Cali, Colombia; 2 Departamento de Investigación y Educación, Clínica de Occidente, Cali 760046, Colombia; 3 Oncóloga Clínica, Clínica de Occidente, Calle 19Nte, 5N–49, Cali 760046, Colombia; 4 Reumatólogo, Clínica de Occidente, Cali 760046, Colombia; 5 Médico Nuclear, Clínica de Occidente, Cali 760046, Colombia; 6 Nefropatólogo, Biomolecular, Bogotá, Colombia

**Keywords:** vasculitis, Henoch-Schönlein purpura, paraneoplastic syndrome

## Abstract

Vasculitides are a set of pathologies that can affect one or several organs, in the short and long term. They are associated with various etiologies, among which paraneoplastic syndromes and medications stand out. Although everyday cancer therapies are more directed against a therapeutic target, their use can cause a wide spectrum of complications. Some treatments against human epidermal growth factor 2 (HER2) have been associated with cutaneous and pulmonary vasculitis. We present the first case of Henoch-Schönlein purpura associated with the use of T-DM1 in a patient with HER2 breast cancer.

## Introduction

Overexpression or amplification of the human epidermal growth factor receptor 2 (HER2) occurs in approximately 20% of all breast cancers, which is associated with more aggressive tumours with a high risk of recurrence and poor prognosis [1]. The availability of target treatments and HER2-directed HER2 antibodies such as: lapatinib, neratinib, trastuzumab, pertuzumab, trastuzumab emtansine (T-DM1) and recently trastuzumab deruxtecan, among others have significantly increased the survival of patients with HER2-positive early and advanced stage breast cancer [2].

The benefits of these treatments have been well studied, but each drug has a different spectrum of complications and toxicities. In the case of T-DM1, the two most frequent side effects are thrombocytopenia and significant elevation of transaminases [3].

Henoch-Schönlein purpura is rare in adults, and maybe the first manifestation as a paraneoplastic syndrome, usually of an occult cancer of the gastrointestinal tract, lung, urinary tract and hematological; however, there are no reports in the literature of this entity associated with the use of T-DM1 [4].

## Case report

We present the case of a 38-year-old woman diagnosed with multicentric left breast carcinoma, hormone receptor-negative, HER2+ stage IIIB by cT3N2M0 since January 2021. Staging studies: contrasted chest computed tomography (CT) scan, contrasted total abdominal CT scan and bone scintigraphy did not show metastatic disease. Due to a family history of breast cancer diagnosed in a cousin younger than 50 years old, a molecular genetic panel NGS 60 genes was performed without finding any alterations.

The patient received neoadjuvant treatment with carboplatin/paclitaxel/ trastuzumab/pertuzumab (TRAIN-2 protocol) for nine cycles between February and July 2021, with partial response. Subsequently, in August 2021, she underwent a modified left radical mastectomy, skin-sparing surgery, nipple-areola complex and lymph node emptying. Pathology showed three foci of invasive non-special type carcinoma. Size of the foci: 1.8, 0.6 and 0.4 cm, uninvolved skin, free margins, 0/18 involved lymph nodes. The patient received adjuvant radiotherapy of 40.5 Gy in 15 fractions in September 2021.

He continued trastuzumab monotherapy from September 2021 for five cycles until November 2021, when he referred recurrent thoracolumbar pain, the spine magnetic resonance imaging (MRI) described a single focal lytic lesion in the anterior aspect of the vertebral body of T11 measuring 9 × 9 mm; however, the total body bone scan did not describe involvement at this level or in other bones. Given the diagnostic uncertainty, positron emission tomography (PET-CT) with fluorodeoxyglucose (FDG) was requested, which detected an extensive solitary lytic lesion in the T11 body; immunohistochemistry of the percutaneous biopsy reported positivity for CKA1/AE3, Gata3, low positivity for Mamaglobin and negativity for LCA and CD138; findings consistent with oligometastatic breast carcinoma. Neurosurgery determined that it was not susceptible to surgical management because there was no unstable fracture or spinal cord compression, so it was ordered to start palliative radiotherapy in this location, with subsequent resolution of the patient’s symptoms. In addition, it was decided to change the treatment to T-DM1 in December 2021, which the patient received with adequate tolerance.

**Figure 1.**
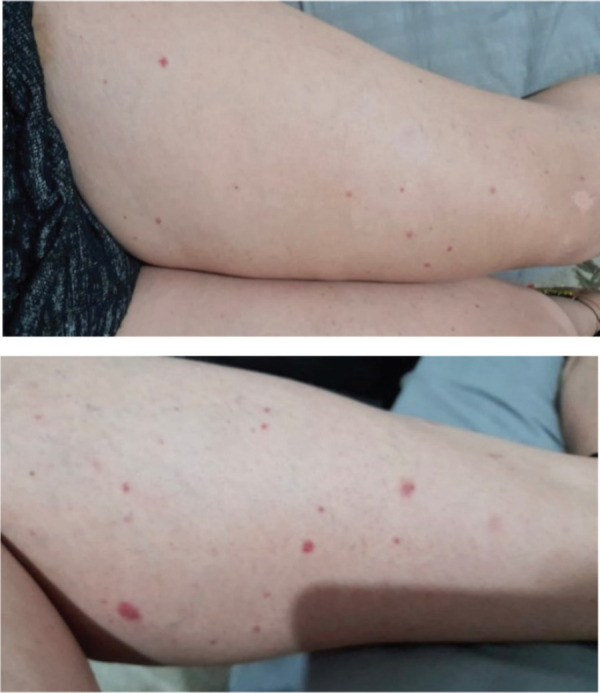
Lower limbs with purpuric lesions at the onset of the clinical picture.

After five cycles of treatment, in March 2022, the patient noted the appearance of purpuric lesions on the lower limbs (Figure 1) and buttocks, occasionally pruritic, without involvement of other body areas. Initially, oncologic dermatology considered a presumptive diagnosis of autoimmune vasculitis versus allergic reaction, so she was managed with moisturising, topical steroid and oral antihistamine, showing partial improvement. One month later he presented hematochezia episode and a routine uroanalysis showed microscopic hematuria and proteinuria in subnephrotic range. A total colonoscopy was requested, which was normal, and a urinary tract CT scan without evidence of alterations of the urinary system, but with the finding of a consolidated pathological fracture of T11, the location where she had been previously irradiated. It was decided to expand studies, an infection and autoimmunity profile was requested, finding positive ANCA in the 1:40 low range, and ANAS in 1:160–1:320, there were no alterations in the hemogram, and she had normal coagulation times (Table 1). She was referred to rheumatology for evaluation. It was noteworthy that the exanthema was variable over time, and its extension and morphology were different in the medical evaluations.

**Figure 2.**
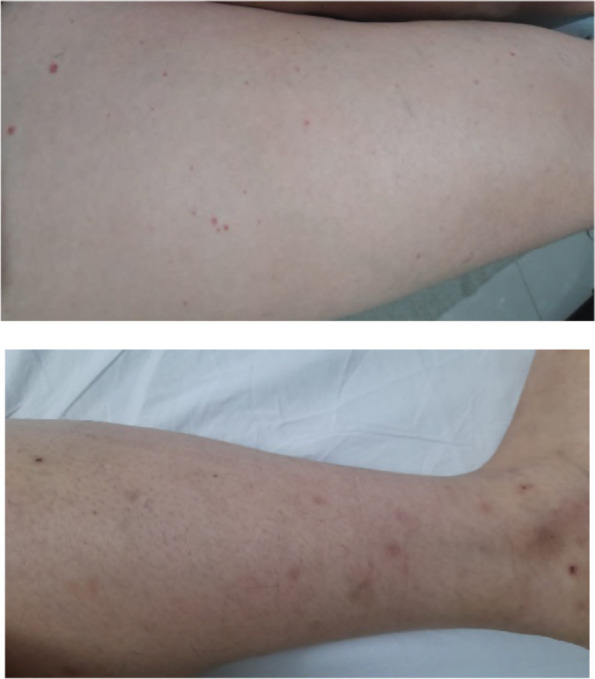
Lower limbs with resolving purpuric lesions.

Five days after the administration of the sixth cycle of T-DM1, the patient presented frank macroscopic hematuria, for which she consulted the emergency department. The case was discussed with the rheumatology department and it was considered that the set of cutaneous, gastrointestinal and urinary manifestations, and the positive autoimmunity profile, could be compatible with IgA vasculitis or Henoch-Schönlein purpura associated with T-DM1; therefore, this drug was suspended. Steroid pulses with methylprednisolone were indicated and on the third day of treatment with the steroid she showed improvement of the cutaneous lesions (Figure 2), as well as urinary and gastrointestinal manifestations, and she did not present any other complications.

**Figure 3.**
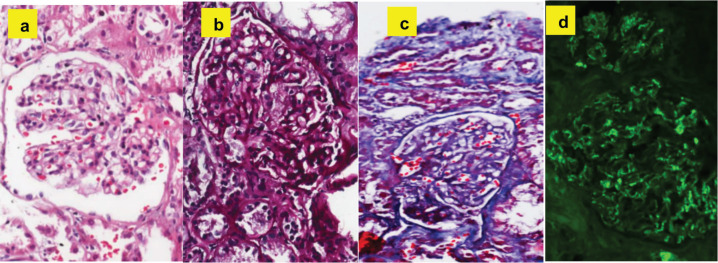
Histopathological findings compatible with the diagnosis of Henoch-Schönlein purpura: (a): Hematoxylin and eosin - expansion of the mesangial matrix with hypercellularity. (b): PAS - expansion of the mesangial matrix. (c): Trichrome - fuscinophilic deposits in mesangium. (d): Immunofluorescence - mesangial positivity for IgA.

Once the methylprednisolone pulses were finished, he continued treatment with oral prednisone at a dose of 1 mg/kg/day and azathioprine 50 mg/day. To confirm the suspected diagnosis, a renal biopsy was ordered, where mesangial IgA deposits were found, compatible with the diagnosis of Henoch-Schönlein purpura (Figure 3).

**Figure 4.**
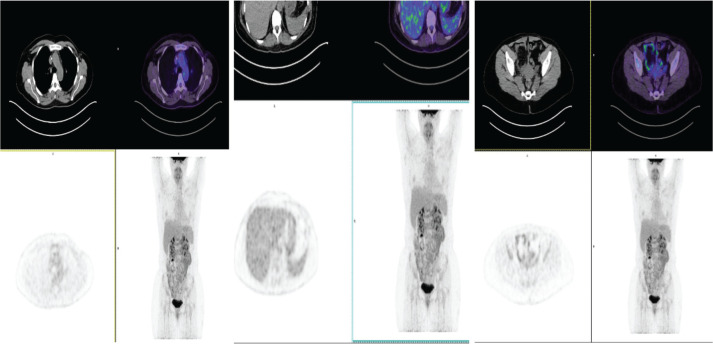
PET-CT of June 2022: no evidence of metabolic changes suggesting local or distant tumour viability. Resolution of oligometastases in T11.

This case was presented to the oncologic medical board supported by rheumatology and an idiosyncratic reaction was conceptualised, possibly to trastuzumab emtansine. It was suggested not to use similar antibodies due to the high risk of relapse of the autoimmune picture. A new PET-CT with re-evaluation of FDG was proposed to define the current status of the disease and to rule out its progression. This was performed in June 2022, where no metabolic changes suggesting local or distant tumour viability were evidenced, with a resolution of oligometastasis in the T11 vertebra (Figure 4).

**Figure 5.**
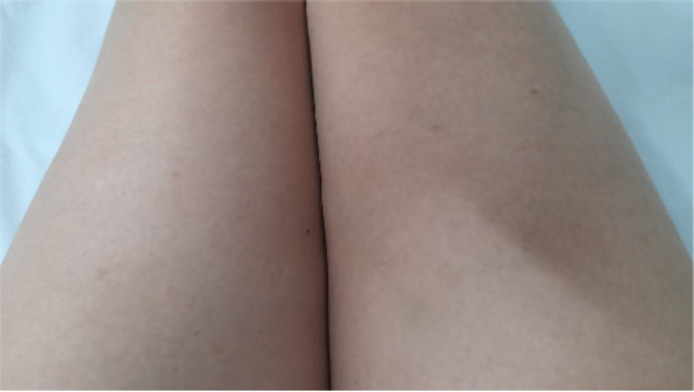
Lower limbs without purpuric lesions.

The patient presented a complete resolution of the purpuric lesions (Figure 5).

She did not continue with another form of HER2 blockade since rheumatology considered that she could be at high risk of presenting a new episode of purpura with severe renal involvement, so she was left on follow-up.

At the last assessment in April 2023, she continued immunosuppressive therapy with prednisolone at 5 mg per day, has remained asymptomatic, and has a complete response to breast cancer.

## Discussion

T-DM1 is an immuno-conjugate composed of trastuzumab, 1 humanized IgG1 monoclonal antibody and emtansine, a microtubule inhibitory cytotoxic agent derived from maytasin [5], linked by a thioether linkage, used in the treatment of HER2-positive breast cancer, as adjuvant management in invasive residual disease and locally advanced unresectable or metastatic disease; in both cases, patients must have received taxane treatment and anti-HER2-targeted therapy [6].

Treatment with T-DM1 is relatively safe and well tolerated, however, side effects have been described that have required dose reduction or have led to discontinuation of treatment; the most frequently described side effect is thrombocytopenia [7] followed by elevation of liver enzymes (AST and ALT), hemorrhage, epistaxis, headache, nausea and fatigue [3].

Regarding vasculitis, one case has been published describing cutaneous vasculitis, in which it was not clear whether it was caused by T-DM1 or by another drug, since the patient was taking several drugs simultaneously [8].

Henoch-Schönlein purpura is an IgA-mediated systemic small vessel vasculitis characterised by palpable non-thrombocytopenic purpura predominantly in the lower limbs (100% of cases), arthralgia or arthritis, abdominal pain, gastrointestinal bleeding, and renal involvement. Biopsy of the affected organ (skin or kidney) demonstrating IgA deposition predominantly supports the diagnosis [9].

Henoch-Schönlein purpura is more common in children, but in adults, it has a more severe course, where renal involvement is one of the most serious manifestations, which determines the long-term prognosis and can present as hematuria, proteinuria even in nephrotic range and in some cases evolve into chronic kidney disease [10]. The etiology is unknown, although it has been related to potential triggering factors such as upper respiratory tract infections or medications such as ß-lactam antibiotics, macrolides, anhypertensives, anticonvulsants and various analgesics [11]. In general, vasculitides are associated with severe inherent hypersensitivity reactions of the individual [12] and are, therefore, not isolated to a particular drug mechanism of action.

This case describes a Henoch-Schönlein purpura caused by T-DM1, whose presentation debuted with renal and gastrointestinal involvement and frank hematuria; in which the renal biopsy, clinical picture, laboratory and imaging tests (including re-evaluation with PET-CT) confirm the diagnosis and rule out the possibility that it was caused by disease progression.

According to the classification of secondary events, the severity of the clinical picture would classify it as grade III (Table 2) [13].

The literature reports that purpura has a higher risk of recurrence when it has gastrointestinal manifestations, has required the use of steroids at baseline, and has renal involvement [14]; being the female sex, a risk factor to present affectation to this last organ. [15] and the presence of hematuria and proteinuria is a predictor of renal failure [16]. Patients with renal involvement relapse after 4–6 weeks and nephritis is more common in adults [17].

Another added factor of severity is that the patient required the use of azathioprine, which is the second-line therapy for HS purpura when patients have not responded to steroids.

Therefore, in case of a relapse of the disease, the patient is contraindicated to receive any anti-HER2 monoclonal antibody, which would negatively affect her prognosis, since her treatment options would be less effective and safe, such as capecitabine, lapatinib, liposomal doxorubicin, vinorelbine, eribulin, ixabepilone, among others.

Currently, the patient is in complete response and has remained asymptomatic from T-DM1 withdrawal under close supervision of clinical oncology (last assessment June 2023), receiving management as indicated by oncologic dermatology and rheumatology.

## Conclusion

It is essential to maintain a safety profile of young patients against new therapies, evaluating and reporting possible new side events of T-DM1, as well as of all systemic drugs prescribed, in order to make an early diagnosis and provide timely treatment, since infrequent side effects represent a limit to the continuity of these therapies.

Institutional pharmacovigilance programs should be strengthened and we should focus our efforts on improving the quality of life and survival of oncology patients.

## Conflicts of interest

Dr Giovanna P Rivas Tafurt has personal fees with Angen and Bristol Myers Squibb, outside the present work. The other authors have no conflicts of interest.

## Funding

This research was not funded by any institution.

## Figures and Tables

**Table 1.** Comparative laboratories during the evolution of purpura.

	Prior to diagnosis		At diagnosis	Control
	In treatment with TDM1			No treatment with TDM1
Laboratories	March/2022	April/2022	May/2022	July/2022
Serum leukocytes	8,660 uL		6,670 uL	7,240 uL
Hemoglobin	13.3 g/dL		14.8 g/dL	13.8 g/dL
Platelets	339,000 µL		228,000 µL	233,000 µL
Serum creatinine	0.53 mg/dL		0.79 mg/dL	0.65 mg/dL
Urea nitrogen			11.2 mg/dL	
Erythrocytes in urine		80 × field	34–45 × field	2–5 × field
Spontaneous proteinuria		100 mg/dL	300 mg/dL	77 mg/dL
Proteinuria in 24H			1.45 g/24 hour	179 mg/24 hour
TTP	19 seconds		27 seconds	25 seconds
TP	10.8 seconds		10.7 seconds	12 seconds
INR	0.94 seconds		0.94 seconds	0.98 seconds
IgA			260.1 mg/dL	
IgM			126.76 mg/dL	
IgG			1,587.41 mg/dL	
ANAS			1:160	
ANCAS			1:40	
Ac Sm			< 2	
Ac SS-A			2.72	
Ac SS-B			< 2	
Ac RNP			< 2	
Anticentromere			Negative	
Complement C3			178.20 mg/dL	142 mg/dL
Complement C4			44.80 mg/dL	22.1 mg/dL
PCR			33.70 mg/L	0.62 mg/L
VSG			30 mm/H	21 mm/H
AST	27 U/L		22 U/L	21 U/L
ALT	32 U/L		16 U/L	27 U/L

**Table 2.** Assessment of the severity of secondary events.

Common terminology criteria for adverse events (CTCAE 5)	
Event	Grade
Purple	3
Hematuria	3
Proteinuria	2
Autoimmune disorders	3
